# Supplementary material for: Optimisation of the Manufacturing Process of Organic-Solvent-Free Omeprazole Enteric Pellets for the Paediatric Population: Full Factorial Design
Source: Pharmaceutics. 2023 Nov 4;15(11):2587. doi: 10.3390/pharmaceutics15112587 (PMC10675058; doi:10.3390/pharmaceutics15112587)
Supplement: Supplementary file 1 [file pharmaceutics-15-02587-s001.zip › pharmaceutics-2654631-supplementary.pdf]

Supplementary Materials: Optimisation of the Manufacturing Process of Organic Solvent-Free Omeprazole Enteric Pellets for the Paediatric Population: Full Factorial Design

Khadija Rouaz-El-Hajoui, Encarnación García-Montoya, Andrea López-Urbano, Miquel Romero-Obon, Blanca Chiclana-Rodríguez, Alex Fraschi-Nieto, Anna Nardi-Ricart, Marc Suñé-Pou, Josep María Suñé-Negre and Pilar Pérez-Lozano

1. Coating parameters

Table S1. Working conditions for the 3 coating layers.

Working Conditions	First Coating Layer	Second Coating Layer	Third Coating Layer
Inlet air temperature	50–60 °C	60–65 °C	55–70 °C
Exhaust air temperature	35–45 °C	30–40 °C	35–45 °C
Product temperature	35–45 °C	35–45 °C	35–45 °C
Würster gun pressure	1.3–2 Bar	1.3–2 Bar	1.3–2 Bar
Pump speed	4–8 rpm	2–4 rpm	5–12 rpm
Compound air outlet position	45–90	60–90	60–90

2. Particle size distribution

MASTERSIZER 2000

Result Analysis Report

**Sample Name:**<br>OMEPRAZOL	**SOP Name:**<br>OMEPARAZOL SCIROCCO	**Measured:**<br>miércoles, 10 de mayo de 2023 8:17:21
**Sample Source & type:**	**Measured by:**<br>User	**Analysed:**<br>miércoles, 10 de mayo de 2023 8:17:22
**Sample bulk lot ref:**<br>Tesi khadija	**Result Source:**<br>Measurement	

**Particle Name:**<br>Polystyrene latex	**Accessory Name:**<br>Scirocco 2000	**Analysis model:**<br>General purpose	**Sensitivity:**<br>Enhanced
**Particle RI:**<br>1.590	**Absorption:**<br>0	**Size range:**<br>0.020 to 2000.000 um	**Obscuration:**<br>2.44 %
**Dispersant Name:**	**Dispersant RI:**<br>1.000	**Weighted Residual:**<br>0.332 %	**Result Emulation:**<br>Off

**Concentration:**<br>0.0002 %Vol	**Span :**<br>2.384	**Uniformity:**<br>1.47	**Result units:**<br>Volume
**Specific Surface Area:**<br>2.13 m²/g	**Surface Weighted Mean D[3,2]:**<br>2.814 um	**Vol. Weighted Mean D[4,3]:**<br>9.745 um	

d(0.1): 1.299 um   d(0.5): 4.872 um   d(0.9): 12.913 um

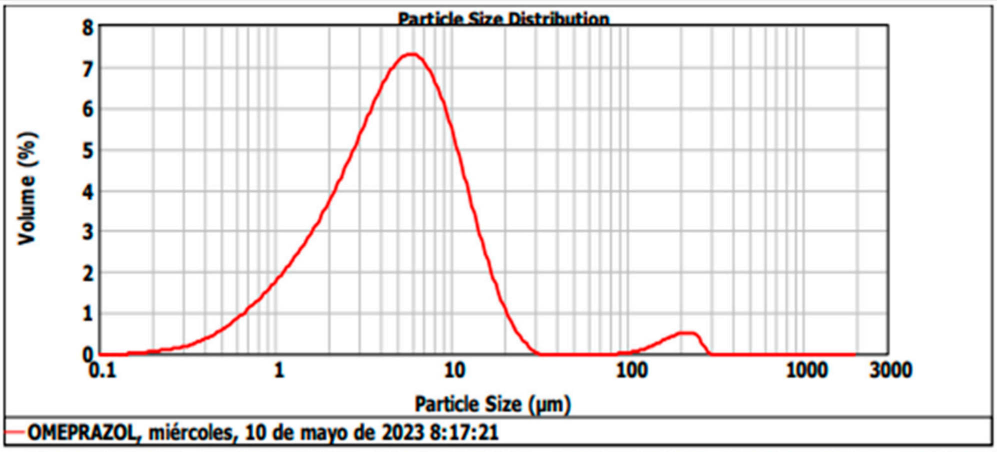

Size (µm)	Vol Under %	Size (µm)	Vol Under %	Size (µm)	Vol Under %	Size (µm)	Vol Under %	Size (µm)	Vol Under %	Size (µm)	Vol Under %
0.010	0.00	0.115	0.00	0.915	5.55	7.287	69.03	51.371	97.98	320.535	100.00
0.011	0.00	0.130	0.00	1.034	6.89	8.233	74.47	58.041	97.98	362.148	100.00
0.014	0.00	0.147	0.00	1.168	8.45	9.302	79.49	65.575	97.98	409.163	100.00
0.018	0.00	0.166	0.01	1.320	10.24	10.510	83.96	70.000	97.98	462.281	100.00
0.021	0.00	0.187	0.04	1.491	12.28	11.874	87.77	74.089	97.98	522.296	100.00
0.027	0.00	0.211	0.08	1.684	14.59	13.416	90.90	83.707	97.98	590.102	100.00
0.030	0.00	0.239	0.14	1.903	17.21	15.157	93.35	94.574	97.98	666.711	100.00
0.034	0.00	0.270	0.23	2.150	20.16	17.125	95.17	98.000	97.98	753.265	100.00
0.038	0.00	0.305	0.35	2.429	23.47	19.348	96.44	106.852	98.00	851.056	100.00
0.043	0.00	0.345	0.51	2.745	27.17	21.860	97.26	120.724	98.05	961.542	100.00
0.049	0.00	0.389	0.73	3.101	31.29	24.698	97.73	136.397	98.16	1086.372	100.00
0.055	0.00	0.440	1.04	3.503	35.83	27.904	97.96	154.104	98.34	1227.408	100.00
0.062	0.00	0.497	1.44	3.958	40.78	31.527	97.98	174.110	98.61	1386.753	100.00
0.070	0.00	0.561	1.97	4.472	46.09	35.620	97.98	196.714	98.98	1566.785	100.00
0.080	0.00	0.634	2.62	5.053	51.69	40.000	97.98	222.251	99.38	1770.189	100.00
0.090	0.00	0.717	3.43	5.709	57.48	40.244	97.98	251.105	99.79	2000.000	100.00
0.102	0.00	0.810	4.40	6.450	63.31	45.469	97.98	283.704	100.00		

Figure S1. PSD of raw material.

3. IR spectrum of the API

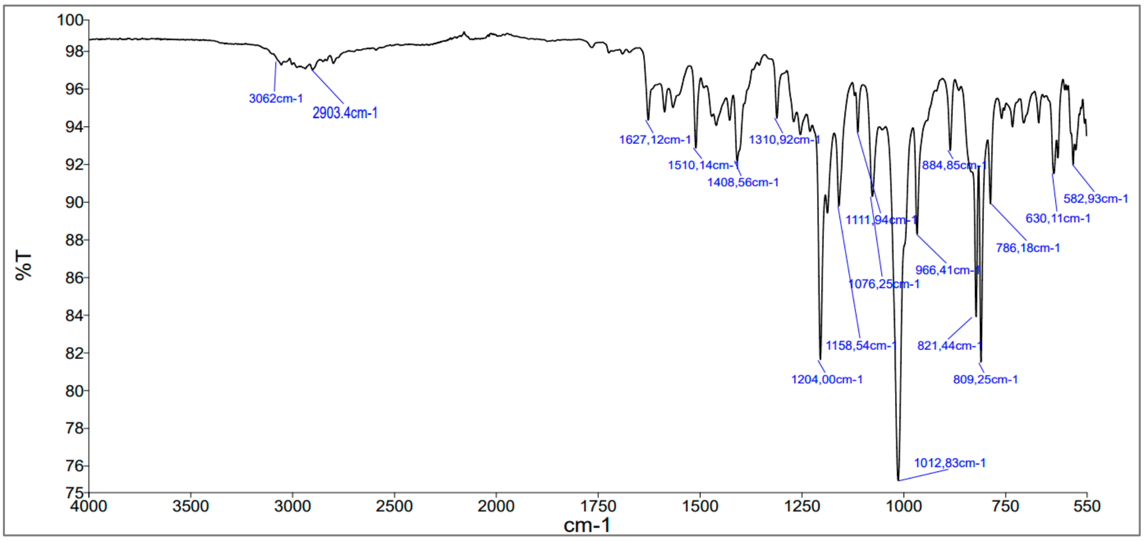

Figure S2. IR spectrum of micronised omeprazole.

4. Differential Scanning Calorimetry analysis

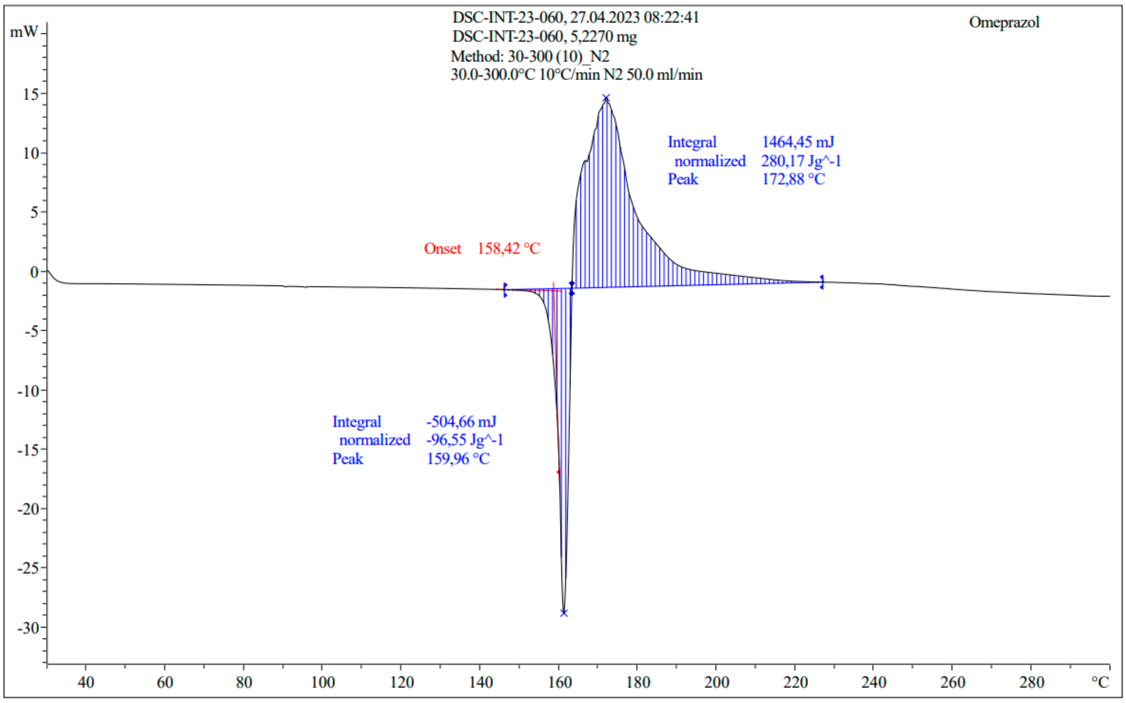

Figure S3. DSC thermogram of micronized omeprazole.

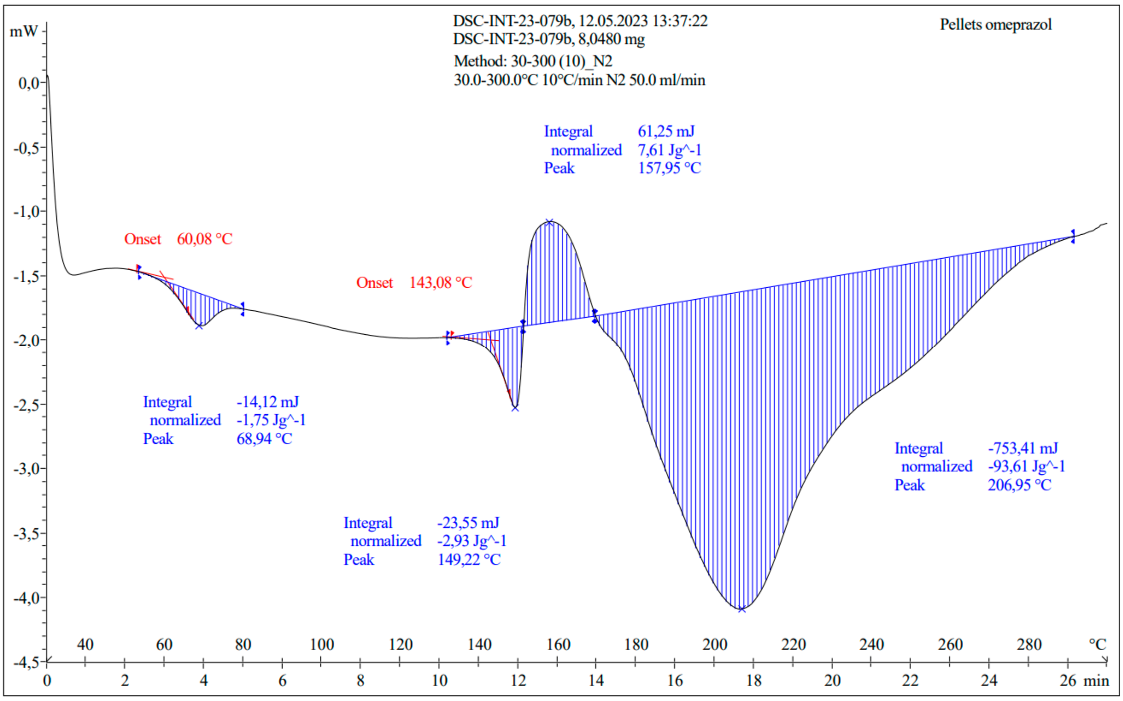

Figure S4. DSC thermogram of omeprazole enteric pellets.

5. X-Ray Diffraction analysis

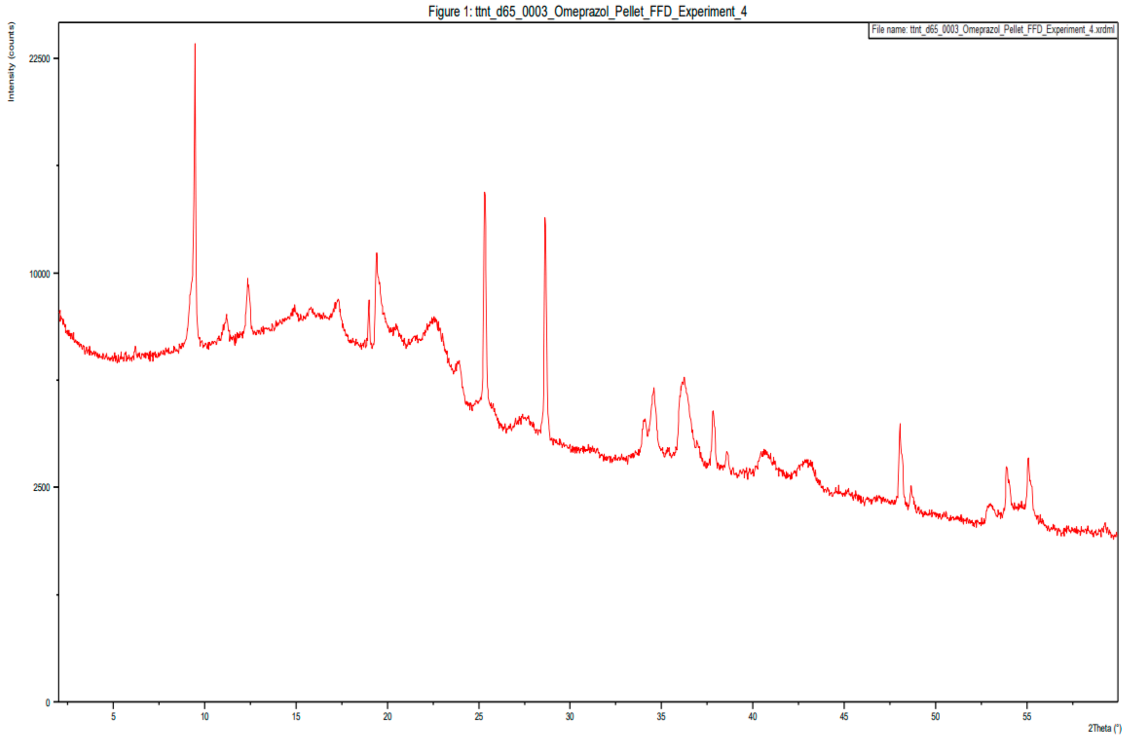

Figure S5. X-ray powder diffraction diagram for NON grinded sample of omeprazole enteric pellets.

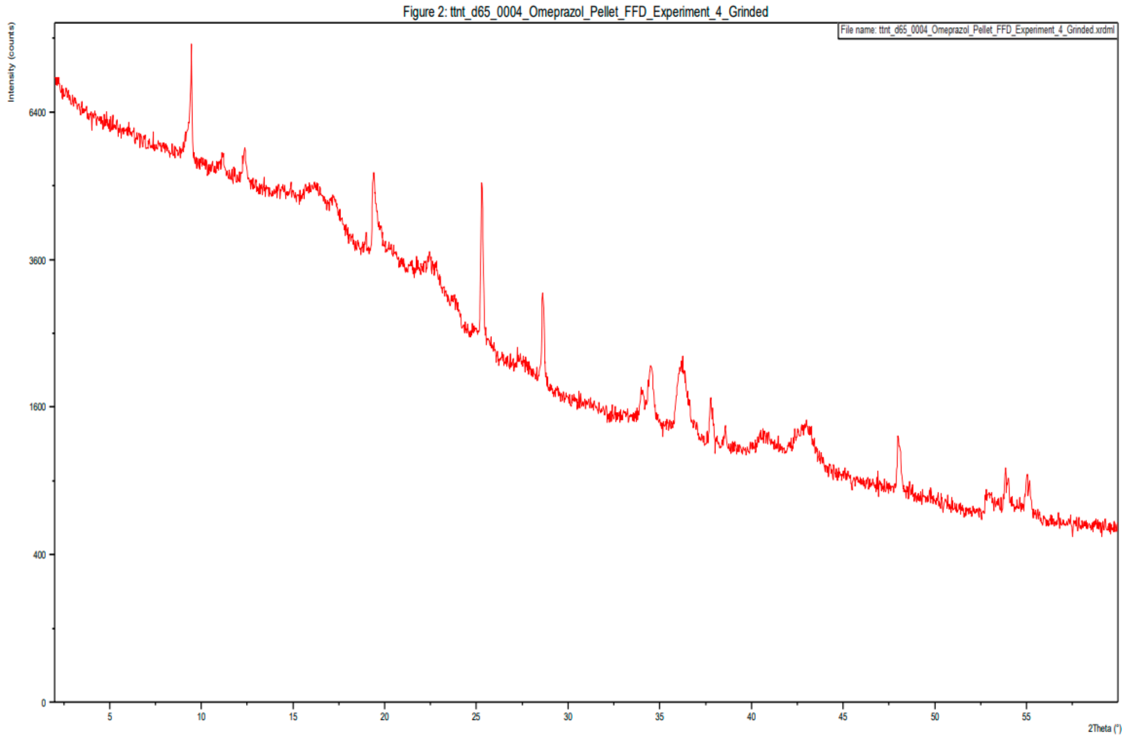

Figure S6. X-ray powder diffraction diagram for grinded sample of omeprazole enteric pellets.
